# Supporting Patients Through Benzodiazepine Tapering: A New Joint Clinical Practice Guideline

**DOI:** 10.1007/s11606-025-09634-z

**Published:** 2025-05-28

**Authors:** Aleksandra E. Zgierska, Maureen P. Boyle, Joseph Conigliaro

**Affiliations:** 1 Departments of Family and Community Medicine, Public Health Sciences, and Anesthesiology and Perioperative Medicine, Penn State College of Medicine, Penn State Milton S. Hershey Medical Center, Hershey, PA USA; 2 American Society of Addiction Medicine, 11400 Rockville Pike, Suite 200, North Bethesda, MD 20852 USA; 3 Department of Medicine, Northwell Health, Donald and Barbara Zucker School of Medicine at Hofstra/Northwell University, New Hyde Park, NY USA

Benzodiazepines are often prescribed for a variety of indications, including anxiety and sleep disorders. Although available clinical guidelines generally recommend avoiding the use of benzodiazepines for more than 4 weeks, long-term use is common.^1^

The effectiveness of benzodiazepine medications may decrease over time as tolerance develops, but risks persist or, in the case of physical dependence and withdrawal, increase over time. While there are some limited indications for longer-term use, such as treatment-resistant anxiety or certain sleep disorders, particularly those associated with abnormal movement, for many patients, the risks may quickly begin to outweigh the benefits.^2,3^

Tapering benzodiazepines can be very challenging, and clinicians across specialties often struggle to help patients reduce the dose or discontinue benzodiazepines without compromising the patient’s wellness or quality of life. It can be particularly difficult in the primary care setting, which has limited capacity for ongoing patient monitoring or providing additional support, such as case management or behavioral treatment. Lack of clear guidance on when to consider tapering and how to approach it has only compounded this issue, limiting some clinicians’ willingness to take on this challenge. Far too often, these combined factors have led to inertia, with patients maintained on benzodiazepine medications long after they stop providing a clinical benefit or begin contributing to harm.

The new *Joint Clinical Practice Guideline on Benzodiazepine Tapering* published in this special issue can assist clinicians with overcoming the dilemmas and uncertainties that have hindered benzodiazepine tapering (when clinically appropriate to do so).^4^ This guideline was developed through a partnership of experts from 10 professional societies representing a broad spectrum of specialties that are commonly involved in prescribing or supporting tapering of benzodiazepines: family medicine, internal medicine, psychiatry, neurology, geriatrics, addiction medicine, obstetrics and gynecology, psychiatric pharmacy, and medical toxicology. The Guideline emphasizes the importance of a patient-centered process with shared decision making and the goal of maximizing benefits while minimizing harm to each person’s health and well-being.

The Guideline recommends regular reassessment of each patient, considering both the risks and benefits of ongoing benzodiazepine therapy as well as those associated with benzodiazepine tapering. Tapering is recommended when the overall risks outweigh the benefits of continued benzodiazepine therapy for the individual. The Guideline also notes that clinicians should generally consider tapering of long-term benzodiazepines in older adults, unless there are compelling reasons for continuation. When benzodiazepine tapering is indicated, the Guideline emphasizes the importance of starting low and going slow (e.g., beginning with a 5–10% reduction in total daily dose every 2–4 weeks), monitoring patients for signs and symptoms of withdrawal or other negative taper-associated effects following each dose reduction, and adjusting the tapering pace based on each patient’s response (i.e., as tolerated by an individual patient).

The Guideline further highlights the importance of managing expectations of both clinicians and patients. The process of tapering long-term benzodiazepine therapy is often slow and non-linear. Although some patients may have no trouble with a relatively fast taper (e.g., 25% daily dose reduction every 2–4 weeks), others may experience significant challenges even with a much slower taper (e.g., 5% daily dose reduction every 4–6 weeks); patient tolerance of the pace of the taper can also change during the process (e.g., with minimal symptoms at the beginning of the taper (i.e., at higher dose), and substantial challenges at the end of the process, at the “tail-end” of tapering). For some patients, especially those who have been taking benzodiazepines for a long time, the tapering process may last for more than a year. They may need to pause at certain points along the way to adjust to the reduced dose. Sometimes, when withdrawal or other symptoms are intolerable, the patient may need to be maintained on a lower, safer dose of benzodiazepines (or, sometimes, even on the original dose) for an extended period or indefinitely.

A panel of patients with lived experience with tapering benzodiazepine medications were engaged throughout the Guideline development process, providing input on the key clinical questions and outcomes of interest for the systematic review, as well as on the Guideline recommendations and narrative discussion. They emphasized the importance of clinician awareness of heterogeneity in patient response to benzodiazepine tapering, and education on how to manage challenges that may arise. In particular, the patient-advisors expressed the hope that clinicians:

- Have awareness of the broad range of potential symptoms of benzodiazepine withdrawal and the risk for protracted withdrawal symptoms, which can last for months or years after the medication has been discontinued;^5,6^
- Know how to support patients who require very slow tapers, for example, by using “microdosing” (or “microtapering”) strategies with liquid benzodiazepine formulations when needed;^6^
- Understand, and help patients understand, the importance of giving time for the brain’s GABA receptors to return to homeostasis (e.g., avoiding the use of alcohol and sedatives, including other benzodiazepine medications and z-drugs (zolpidem, zopiclone, and zaleplon)).

It is important to emphasize that physical (or physiological) dependence is distinct from a benzodiazepine use disorder (BUD). Nearly all patients who take benzodiazepines daily or near daily for a prolonged period (ie, more than a month) will develop physical dependence, which manifests in tolerance and risk for withdrawal when the medication is stopped, or the dose is reduced. Benzodiazepine withdrawal can manifest with a wide array of symptoms that can sometimes be difficult to distinguish from recurrence of the condition for which the medication was originally prescribed. It is important for clinicians and patients to be aware of the heterogeneous presentations of benzodiazepine withdrawal symptoms, including protracted withdrawal, and not to minimize or dismiss the concerns of patients who are experiencing less-common symptoms.

It is estimated that only 1.5% of individuals who are treated with benzodiazepines develop a BUD,^7^ and patients with BUD typically need additional services and support during benzodiazepine tapering. Clinicians should consider referring patients with BUD to a qualified addiction treatment provider so that benzodiazepine tapering can be managed by or in coordination with the specialty team.

It is critical for clinicians to demonstrate and maintain compassion and understanding throughout the tapering process, starting during the initial conversations with patients about tapering the medication. Patients often need reassurance from their clinicians and to know they will be supported and listened to throughout the process. Clinicians and patients should expect that patients will provide input on and, when appropriate, influence the tapering strategy.

Benzodiazepine tapering is challenging for physicians and advanced practice providers across specialties. However, as most benzodiazepines are prescribed in primary care settings, this guideline is particularly important for family medicine and internal medicine clinicians. A number of resources have been developed to help clinicians implement the recommendations in the Guideline, including continuing medical education, clinician and patient pocket guides and handouts, and downloadable clinical tools such as a benzodiazepine dose equivalents and decision flow charts.^8^

As we saw after the release of the CDC Guideline for Prescribing Opioids for Chronic Pain in 2016, Guidelines can have unintended consequences.^9^ There may be a large population of patients for whom benzodiazepine tapering is indicated. Clinicians should prioritize those who are at the highest risk of harm. Benzodiazepine medications should not be abruptly discontinued in patients who are physically dependent. Alternative strategies for managing benzodiazepine withdrawal risk (e.g., tapering with very long-acting agents) may be used when there are compelling reasons for more rapid taper or discontinuation and are also discussed in the Guideline. This Guideline should not be used as a reason to abandon patients who require a benzodiazepine taper or continued long-term benzodiazepine therapy.

While considerations for initiating benzodiazepine were outside of the scope of this Guideline, it is clear we need to think carefully about when to start these medications and particularly when to continue with subsequent prescriptions. It is critical that we educate patients on these risks at the outset to support truly informed consent.

## Data Availability

All data analyzed during the development of the guideline are available in that article, published in this supplement.
